# Methylphenidate and the risk of burn injury among children with attention-deficit/hyperactivity disorder

**DOI:** 10.1017/S2045796020000608

**Published:** 2020-07-20

**Authors:** Vincent Chin-Hung Chen, Yao-Hsu Yang, Ting Yu Kuo, Mong-Liang Lu, Wei-Ting Tseng, Tsai-Yu Hou, Jia-Ying Yeh, Charles Tzu-Chi Lee, Yi-Lung Chen, Min-Jing Lee, Michael E. Dewey, Michael Gossop

**Affiliations:** 1 Department of Psychiatry, Chang Gung Memorial Hospital, Chiayi Branch, Chiayi, Taiwan; 2 School of Medicine, Chang Gung University, Taoyuan, Taiwan; 3 Health Information and Epidemiology Laboratory, Chang Gung Memorial Hospital, Chiayi, Taiwan; 4 Department of Traditional Chinese Medicine, Chang Gung Memorial Hospital, Chiayi Branch, Chiayi, Taiwan; 5 Department of Psychiatry & Psychiatric Research Center, Wan-Fang Hospital and Taipei Medical University, Taipei, Taiwan; 6 Department of Psychiatry, School of Medicine, College of Medicine, Taipei Medical University, Taipei, Taiwan; 7 Department of Psychiatry, Chang Gung Memorial Hospital, Kaohsiung Branch, Chiayi, Taiwan; 8 Department of Health Promotion and Health Education, National Taiwan Normal University, Taipei, Taiwan; 9 Department of Healthcare Administration, Asia University, Taichung, Taiwan; 10 Department of Psychology, Asia University, Taichung, Taiwan; 11 Institute of Psychiatry, Psychology and Neuroscience, King's College London, London, UK; 12 National Addiction Centre, Institute of Psychiatry Psychology and Neuroscience, King's College London, London, UK

**Keywords:** Attention-deficit/hyperactivity disorder, burn injury, methylphenidate

## Abstract

**Aims:**

Attention-deficit/hyperactivity disorder (ADHD) is associated with a higher risk of burn injury than in the normal population. Nevertheless, the influence of methylphenidate (MPH) on the risk of burn injury remains unclear. This retrospective cohort study analysed the effect of MPH on the risk of burn injury in children with ADHD.

**Method:**

Data were from Taiwan's National Health Insurance Research Database (NHIRD). The sample comprised individuals younger than 18 years with a diagnosis of ADHD (*n* = 90 634) in Taiwan's NHIRD between January 1996 and December 2013. We examined the cumulative effect of MPH on burn injury risk using Cox proportional hazards models. We conducted a sensitivity analysis for immortal time bias using a time-dependent Cox model and within-patient comparisons using the self-controlled case series model.

**Results:**

Children with ADHD taking MPH had a reduced risk of burn injury, with a cumulative duration of treatment dose-related effect, compared with those not taking MPH. Compared with children with ADHD not taking MPH, the adjusted hazard ratio for burn injury was 0.70 in children taking MPH for <90 days (95% confidence interval (CI) 0.64–0.77) and 0.43 in children taking MPH for ≥90 days (95% CI 0.40–0.47), with a 50.8% preventable fraction. The negative association of MPH was replicated in age-stratified analysis using time-dependent Cox regression and self-controlled case series models.

**Conclusion:**

This study showed that MPH treatment was associated with a lower risk of burn injury in a cumulative duration of treatment dose-related effect manner.

Attention-deficit/hyperactivity disorder (ADHD) is a common neurodevelopmental disorder characterised by inattention, hyperactivity, impulsivity and cognitive dysfunction (Agerbo *et al*., 2002). The global lifetime prevalence of ADHD is estimated to be 7.2% (Thomas *et al*., 2015). ADHD is significantly associated with impairments in daily life, daily functioning at school or work, and social and emotional development (Buitelaar and Medori, 2010). Furthermore, it has implications for family stress and health care resource consumption (Dittmann *et al*., 2014). In addition, the core symptoms of ADHD, including inattention, distractibility and impulsivity, probably account for the increased risk of unintentional injuries (Cairney, 2014). ADHD is associated with an increased risk of accident and injury (Rowe *et al*., 2004; Chang *et al*., 2014; Chang *et al*., 2019), leads to a shorter life expectancy, and those accidents or injuries are the most common cause of death in ADHD patients (Dalsgaard *et al*., 2015b; Chen *et al*., 2019).

Burn injuries can be a devastating event, imposing a physical, psychological and economic burden on such patients and society (Chen *et al*., 2014b). Children with ADHD are at an increased risk of burn injury (Mangus *et al*., 2004; Thomas *et al*., 2004; Fritz and Butz, 2007; Badger *et al*., 2008; Ghanizadeh, 2008). The most recent case–control study including 223 patients demonstrated that the prevalence of burn injury among children with ADHD (10.6%) was higher than that among the control group (2.0%) (Ghanizadeh, 2008). A retrospective study by Badger *et al*. also reported that children with ADHD or ADD were more frequently involved in activities with a high risk of burn injury and more frequently experienced mental health difficulties (Badger *et al*., 2008).

Pharmacotherapy is the first-line treatment for children with ADHD, and it can effectively reduce ADHD symptoms at all ages (Subcommittee on Attention-Deficit/Hyperactivity *et al*., 2011). Robust evidence has been reported to support methylphenidate (MPH) as preferred first-choice medications for the short-term treatment of ADHD in children and adolescents (Cortese *et al*., 2018). Studies have proposed that treatment with stimulants might reduce the risk of injuries in patients with ADHD (Dalsgaard *et al*., 2015a; Man *et al*., 2015; Mikolajczyk *et al*., 2015; Liang *et al*., 2017). One population-based prospective cohort study reported that treatment with ADHD drugs reduced the risk of injuries and emergency ward visits (Dalsgaard *et al*., 2015a). No study has evaluated whether exposure to psychostimulant treatment mitigates the risk of burn injury or examined the effect of the duration of medication administration. We therefore explored the effect of the psychostimulant MPH on the risk of burn injury in patients with ADHD and whether the mitigating effects are related to the duration of exposure, by using a nationwide population-based dataset in Taiwan. We hypothesised that ADHD medication was linked to a lower risk of burn injury.

## Materials and methods

### Study design and participants

In this retrospective cohort study, we used data from the Taiwan National Health Insurance Research Database (NHIRD) under the aegis of the National Health Research Institute, which includes data on outpatient, ambulatory and hospital inpatient care, as well as on dental services. Taiwan launched a single-payer National Health Insurance programme on 1 March 1995, covering the delivery of all health care services to 99.5% of the national population (Ng, 1997). The NHIRD holds some important information, such as patients' demographic data, the medical institution visited, diagnostic codes, the drugs prescribed, the date of any prescriptions given and any claimed medical expenses. The database has been used in many epidemiologic studies in Taiwan (Lee *et al*., 2017; Chen *et al*., 2018). Several validation studies have shown that the data set represents modest to high sensitivity and positive predictive values (Hsieh *et al*., 2019).

The ADHD cohort was selected from NHIRD. It included individuals younger than 18 years by 31 December 2013. For ensuring the diagnosis of ADHD, we only recruited participants with ADHD who received at least one inpatient diagnosis of ADHD (International Classification of Disease, 9th revision [ICD-9] code: 314) or more than two outpatient diagnoses within 1 year between 1 January 1996 and 31 December 2011.

### Outcomes

The main outcome of this study was burn injury (ICD-9-CM codes: 940–949). Patients with a diagnosis of burn injury (Mangus *et al*., 2004) before the diagnosis of ADHD were excluded. Patients with ADHD were followed up for the incidence of burn injury as an outcome, or until age 18, or to the end of 2013.

### Assessment of other characteristics

Several covariates were selected, including sex, age, urbanisation level of residence and seizure (ICD-9 code: 345). Psychiatric comorbidity included intellectual disability (ICD-9 codes: 317–319), autism (ICD-9 code: 299), conduct disorder (ICD-9 code: 312), opposition defiant disorder (ICD-9-CM code: 313.81), anxiety (ICD-9 code: 300) and depression (ICD-9 codes: 296.2, 296.3, 300.4 and 311). Baseline medication use was identified based on a prescription of any benzodiazepine (ATC codes: N03AE, N05BA and N05CD), benzodiazepine-related drugs (ATC code: N05CF), antipsychotic (ATC code: N05A) or antidepressant (ATC code: N06A) 12 months prior to the burn injury diagnosis.

MPH (ATC code: N06BA04) has been the only stimulant approved for treating ADHD in Taiwan and is regarded as the first-line treatment for ADHD according to Taiwan's National Insurance. In 2017, atomoxetine (ATX), a non-stimulant, was also approved for ADHD treatment in Taiwan. However, compared with MPH, ATX has a much lower prescription rate (4% in all patients with ADHD) (Lee *et al*., 2016). ATX is recommended only for cases where patients have insufficient treatment outcomes (i.e. inefficacy and intolerability) from MPH. Thus, it can be reasonably assumed that patients who received ATX have had prior MPH treatment exposure. Therefore, we included patients with only MPH exposure in this study. We investigated the treatment duration effect by examining MPH use in the ADHD cohort. The definition of treatment duration was the cumulative length of MPH exposure (days) within the follow-up period until end-points of burn injury, death or the end of the study. The ADHD population was divided into three subgroups based on the duration of MPH prescription: 0, <90 and ≥90 days. This study was reviewed and approved by the Institutional Review Board of Chang Gung Memorial Hospital.

### Statistical analysis

All data management and statistical analyses were performed using SAS Version 9.4 (SAS Institute Inc., Cary, NC, USA) and R 4.0.0 (R Foundation for Statistical Computing, Vienna, Austria). To describe the distribution of the study population, a *χ*^2^ test was used to compare the characteristics between the ADHD and control groups. The adjusted risk of burn injury between ADHD with and without MPH medication and the preventable fraction of MPH medication among individuals with ADHD was estimated using a Cox proportional hazards regression model. The results are presented as hazard ratios (HRs) with 95% confidence intervals (CIs). We calculated the duration of medication use for each patient with ADHD and tested the cumulative effect on the burn incidence according to stratification into three groups, 0, <90, ≥90 days, by adjusting for covariates in competing risk-adjusted Cox regression models (Allgulander and Fisher, 1986). Age-stratified analysis was also conducted to examine the cumulative effect of MPH at three different age groups: (1) age <6, (2) age between 6 and 12, and (3) age >12 and <18. Participants' age is calculated by the difference between their birthdate and the end of follow-up (i.e. 31 December 2013) (Allgulander and Fisher, 1986). To examine the immortal time bias, a sensitivity analysis using time-dependent Cox hazards regression analysis was performed. We also estimated the preventable fraction: the proportion of incidents of burn in patients with ADHD not taking MPH that could be prevented by MPH medication.

**Fig. 1.**
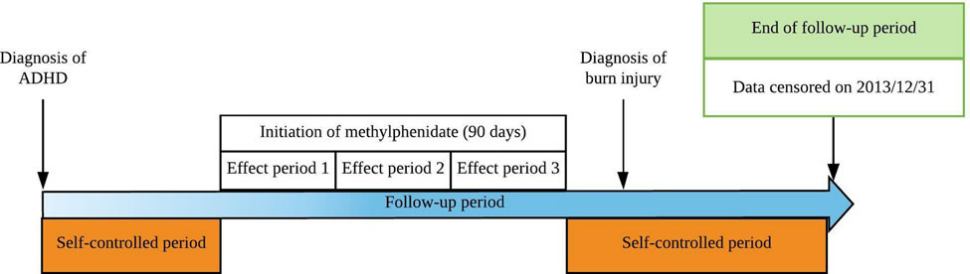
Overview of the self-controlled case series study design. The self-controlled case series study design was used in this study for within-patient comparisons to examine the effect of methylphenidate on traumatic brain injury in adolescent patients with ADHD. The effect period for methylphenidate is set at 1–3 months. Thus, the effect period was split into three 1-month effect periods: 0–30, 31–60 and 61–90 days at the end of each treatment period. Individual's time not within the treatment period would serve as a self-controlled period for self-comparison. Time point of cohort entry was defined as the date of age of onset of ADHD. The end of follow-up was 31 December 2013.

For within-patient comparisons, we used the self-controlled case series (SCCS) model, in which time was divided into periods similar to the stratified Cox regression model, with each patient as a separate stratum (i.e. the patient served as his or her own control). The risk estimated from the SCCS model indicates the risk of burn injury when individuals took MPH compared to the period when they did not take MPH. The SCCS model automatically adjusts for all time-invariant factors (e.g. sex) for the same patient before and during the follow-up. The effect period for MPH was set at 1–3 months based on previous studies (Chen *et al*., 2014a; Chang *et al*., 2016). Thus, the effect period was split into three 1-month effect periods: 0–30, 31–60 and 61–90 days from the end of each treatment period. Finally, we combined the three effect periods into one to report the average pooled estimate of MPH use for burn injury. Figure 1 is present to illustrate the SCCS study design in this cohort. The SCCS model was applied using the SCCS package in R (Farrington *et al*., 2018). Furthermore, a moderation analysis based on the SCCS model was conducted to examine whether the neurodevelopment disorders (i.e. intellectual disability and autistic spectrum disorder) modify the effect of MPH on burn injury in children and adolescents with ADHD.

## Results

Table 1 shows the characteristics of children with ADHD stratified to three subgroups: 0 days (22 347 patients), <90 days (17 766 patients) and ≥90 days (50 521 patients). The risk of burn injury was 6.7, 4.5 and 2.9% in the three groups, respectively (*p* < 0.0001). The preventable fraction of MPH medication was 0.508, indicating that 50.8% (11 352 patients) incidence of burn injury could be prevented if they took MPH medication.

**Table 1.** Characteristics of children with ADHD with and without MPH use

Variables	Frequency		MPH = 0 day		0–90 days		≥90 days		
	*N*	%	*N*	%	*N*	%	*N*	%	*p* value
Total	90 634		22 347		17 766		50 521		
Gender									
Female	18 512	20.4	5550	24.8	3863	21.7	9099	18.0	<0.0001
Male	72 122	9.6	16 797	75.2	13 903	78.3	41 422	82.0	
Age									
0–5	20 372	22.5	9157	41.0	3016	17.0	8199	16.2	<0.0001
6–11	62 880	69.4	11 951	53.5	12 738	71.7	38 191	75.6	
12–18	7382	8.1	1239	5.5	2012	11.3	4131	8.2	
Covariates									
Seizure	4067	4.5	1175	5.3	732	4.1	2160	4.3	<0.0001
Intellectual disability	431	0.5	161	0.7	83	0.5	187	0.4	<0.0001
Autism	8603	9.5	3042	13.6	1523	8.6	4038	8.0	<0.0001
Conduct disorder	3452	3.8	724	3.2	641	3.6	2087	4.1	<0.0001
Opposition defiant disorder	5132	5.7	280	1.3	725	4.1	4127	8.2	<0.0001
Anxiety	18 528	20.4	2115	9.5	3025	17.0	13 388	26.5	<0.0001
Depression	2368	2.6	373	1.7	459	2.6	1536	3.0	<0.0001
Benzodiazepine use	3045	3.4	673	3.0	615	3.5	1757	3.5	0.0039
Z-drugs use	165	0.2	22	0.1	30	0.2	113	0.2	0.0011
Antipsychotics use	5340	5.9	849	3.8	828	4.7	3663	7.3	<0.0001
Antidepressants use	2765	3.1	405	1.8	456	2.6	1904	3.8	<0.0001
Urbanised level of residence									
1 (City)	32 557	35.9	9058	40.5	5962	33.6	17 537	34.7	<0.0001
2	43 111	47.6	10 142	45.4	8685	48.9	24 284	48.1	
3	10 246	11.3	2203	9.9	2189	12.3	5854	11.6	
4 (Villages)	4720	5.2	944	4.2	930	5.2	2846	5.6	
Burn injury	3742	4.1	1494	6.7	792	4.5	1456	2.9	<0.0001

Table 2 shows the influences of the MPH cumulative effect on the burn risk among patients with ADHD and different age groups. For the whole sample, compared with patients with ADHD not taking MPH, those taking MPH for <90 days had an adjusted HR of 0.70 for burn injury (CI 0.64–0.77) and those taking MPH for ≥90 days had an adjusted HR of 0.43 (95% CI 0.40–0.47). The results of the trend test were significant (*p* < 0.01). In addition, age-stratified analyses revealed a similar pattern in different age groups and the adjusted HR ranged from 0.55 to 0.87 for those taking MPH for <90 days and 0.32 to 0.55 for those taking MPH for ≥90 days, respectively. Table 3 shows the sensitivity analysis which confirmed the inverse association between MPH and risk of burn injury using the time-dependent model.

**Table 2.** Cox proportional hazards model for burn injury in children with ADHD and age-stratified analysis

Methylphenidate use	Unadjusted analysis			Adjusted analysis model^a^		
	HR	95% CI	*p* value	HR	95% CI	*p* value
Whole sample						
0 day (ref.)	1.00	–	–	1.00	–	–
<90 days	0.65	0.60–0.71	<0.0001	0.70	0.64–0.77	<0.0001
≥90 days	0.39	0.37–0.42	<0.0001	0.43	0.40–0.47	<0.0001
Age < 6						
0 day (ref.)	1.00	–	–	1.00	–	–
<90 days	0.54	0.46–0.63	<0.0001	0.55	0.46–0.64	<0.0001
≥90 days	0.31	0.27–0.35	<0.0001	0.32	0.28–0.37	<0.0001
6 ≤ age < 12						
0 day (ref.)	1.00	–	–	1.00	–	–
<90 days	0.88	0.78–0.99	0.0278	0.87	0.78–0.98	0.0213
≥90 days	0.53	0.48–0.59	<0.0001	0.54	0.48–0.60	<0.0001
12 ≤ age < 18						
0 day (ref.)	1.00	–	–	1.00	–	–
<90 days	0.83	0.54–1.28	0.3983	0.84	0.55–1.31	0.4443
≥90 days	0.55	0.37–0.82	0.0038	0.55	0.37–0.83	0.0045

a Adjusted by seizure, intellectual disability, autism, conduct disorder, opposition defiant disorder, anxiety, depression and psychotropic use (benzodiazepine, Z-drugs, antipsychotics and antidepressants).

**Table 3.** Time-dependent Cox proportional hazards model for burn injury in children with ADHD

	Unadjusted analysis			Adjusted analysis model^a^		
Variables	HR	95% CI	*p* value	HR	95% CI	*p* value
Methylphenidate						
0 DDD (ref.)	1.00	–	–	1.00	–	–
>0 day	0.65	0.61–0.70	<0.0001	0.72	0.67–0.78	<0.0001

a Adjusted by seizure, intellectual disability, autism, conduct disorder, oppositional defiant disorder, anxiety, depression and psychotropic use (benzodiazepine, Z-drugs, antipsychotics and antidepressants).

Table 4 presents the effect of MPH on burn injury within patients with ADHD, which was determined using the SCCS model. Within-patient comparisons revealed a significant reduction in the risk of burn injury in effect periods from 0 to 30 (HR 0.49, 95% CI 0.44–0.55), 30 to 60 (HR 0.46, 95% CI 0.40–0.54) and 60 to 90 days (HR 0.51, 95% CI 0.42–0.41). An average pooled estimate of these effect periods indicated an HR of 0.49 and 95% CI of 0.45–0.53. Furthermore, we found that the effect of MPH on burn injury was attenuated in participants with ADHD and intellectual disability (*p* = 0.002), but not in autistic spectrum disorder (*p* > 0.05). The protective effect (RR) was decreased from 0.49 in participants with ADHD to 0.61 in participants with ADHD and intellectual disability.

**Table 4.** Stratified Cox regression for self-controlled case series model of burn injury in children with ADHD

Variables	Adjusted analysis model^a^		
	HR	95% CI	*p* value
Effect period of methylphenidate			
0–30 days	0.49	(0.44–0.55)	<0.0001
30–60 days	0.46	(0.40–0.54)	<0.0001
60–90 days	0.51	(0.42–0.61)	<0.0001
0–90 days	0.49	(0.45–0.53)	<0.0001

a Hazard ratios were calculated using stratified Cox regression for SCCS model, which automatically adjusts for all time-invariant factors (e.g. sex) for the same patient before and during the follow-up.

## Discussion

To our knowledge, this is the first nationwide population-based cohort study to investigate the effect of medication usage on the risk of burn injury in ADHD patients. Our study looked into the risk of burn injury specifically and added to the existing evidence that MPH treatment was associated with a lower risk of burn injury among ADHD children. We found a lower incidence of burn injury in patients with ADHD prescribed MPH in the whole sample and different age subgroups. In total, 50.8% incidence of burn injury among patients with ADHD not taking MPH could be prevented if they took MPH medication. The effect was more prominent with increased duration of MPH use: a 30% reduction in the risk of burn injury with <90 days of MPH use was observed, and a 57% reduction in the risk of burn injury with ≥90 days of MPH use was noted. Similar reductions were replicated in age-stratified analysis and the time-dependent and SCCS design. These results suggest the robust effect of MPH use on burn injury in ADHD.

Research has indicated that ADHD patients have a higher risk for burn injury than non-ADHD controls (Fredriksen *et al*., 2014). The potential explanations include impulsivity, attention-deficit-related carelessness, overlooking danger, impairment in executive function and motor coordination. This study found that ADHD patients prescribed MPH had a reduced likelihood of burn injury and prior studies have also demonstrated correlations between burn injury and MPH. One retrospective chart review examined children admitted to a burn care facility with respect to ADHD diagnosis or history of stimulant prescription and found that although a high percentage of these children (nearly 89%) had been prescribed stimulant medication, approximately 42% of them had not taken their usual dose of MPH on the day of the burn injury; they were injured by impulsive fire-play behaviour (Thomas *et al*., 2004). In the present study, an average within-patient risk reduction of 51% was noted from comparing periods of time exposed to stimulant medication and unexposed periods. We expand on prior findings that MPH treatment plays a role in the reduced risk of burn injury in patients with ADHD. Several studies have suggested benefits on core symptoms and cognitive performance improvement after stimulant treatment (Fredriksen *et al*., 2014; Setyawan *et al*., 2015). Nevertheless, the exact mechanism of such stimulants underlying reduction of burn injury risk in children with ADHD requires further investigation.

We also investigated the cumulative effect of MPH (using the cumulative duration of MPH prescription) on the risk of burn injury in patients with ADHD and found that the burn injury risk reduction was linked to an increased MPH usage duration. Studies have identified attenuation in the risk of fracture, suicide and traumatic brain injury to be associated with the dosage effect of MPH treatment (Chen *et al*., 2017; Liang *et al*., 2017; Liao *et al*., 2018). Long-term stimulant treatment normalises delayed brain development and yield to neuroadaptive and neurochemical change (Schweren *et al*., 2013). Functional magnetic resonance imaging studies have also shown that exposure to psychostimulants may alter brain function connectivity and activity (van der Marel *et al*., 2015). Our findings extend previous evidence and hold implications for clinical practice – sufficient duration of MPH treatment may be protective against burn injury.

This nationwide population-based study has several strengths. First, the nationally representative sample was substantial and minimised selection bias. Second, patients with ADHD were identified through physician-based diagnoses. Third, all MPH prescriptions are recorded in the NHIRD, avoiding misclassification bias. Also, by excluding burn injuries prior to ADHD diagnosis, the reverse causal relationship between ADHD and burn injury was eliminated. Finally, we used different models to demonstrate the robust treatment effect of MPH on burn injury. The between-subject treatment effect was observed in the Cox proportional hazards models. We found a significant cumulative duration of treatment dose-related effect of MPH treatment on burn risk, whereas within-subject treatment effects were assessed using the SCCS model. Some studies have also used the within-subject methodology (i.e. SCCS or case-crossover study design) to examine the effect of MPH on unintentional injuries (Ruiz-Goikoetxea *et al*., 2018); however, these studies suffered from the small sample size because their sample size merely ranged from 328 to 4934 (Ruiz-Goikoetxea *et al*., 2018). As a result, they focused on any type of injury, and they were not able to examine some specific causes of injury. For example, in Raman *et al*.'s study, they had 328 individuals with ADHD who experienced an incident medically attended injury event and received at least one prescription for stimulant medication, but only 1.2% (4/328) reported experiencing burn injury (Raman *et al*., 2013). In our study, because of the enforcement rules of the National Health Insurance in Taiwan, citizens in Taiwan are required to enrol in this insurance programme, resulting in sufficient data for examining this issue. In terms of the SCCS, we revealed that the risk was significantly reduced during the possible effect period of MPH treatment (1–3 months after treatment initiation). In addition, the major advantage of the SCCS model was that indication bias and any time-invariant confounders would be automatically controlled because of self-comparison. Finally, the time-dependent Cox model was conducted to address immortal time bias, which refers to a period of follow-up duration, by design, the outcome cannot occur. All these models provided similar results, suggesting that our study provided robust estimates of the inverse effects of MPH treatment on burn risk in children with ADHD.

This study had several limitations. First, the accuracy of disease diagnoses for ADHD or burn injury has not been documented. Second, information on drug adherence was lacking. Third, we did not evaluate the therapeutic effect of non-pharmacological treatment because behavioural therapy and psychoeducation were not fully registered in the NHIRD. Finally, the lack of MPH use in some patients may be due to a worsening of symptoms, and we did not have information on the severity of ADHD.

In conclusion, MPH treatment was linked to a lower risk of burn injury in children with ADHD, and the effect was stronger with longer MPH use.

## Data Availability

These data were owned by the National Health Research Institutes. The present study was based on the National Health Insurance Research Database provided by the Central Bureau of National Health Insurance, the Department of Health, and managed by the National Health Research Institutes.
